# A systematic review and network meta-analysis comparing azacitidine and decitabine for the treatment of myelodysplastic syndrome

**DOI:** 10.1186/s13643-018-0805-7

**Published:** 2018-09-19

**Authors:** Jehad Almasri, Hassan B. Alkhateeb, Belal Firwana, Mohamad Bassam Sonbol, Moussab Damlaj, Zhen Wang, M. Hassan Murad, Aref Al-Kali

**Affiliations:** 1 Evidence-Based Practice Research Program, Mayo Clinic, Rochester, MN USA; 2 Robert D. and Patricia E. Kern Center for the Science of Health Care Delivery, Rochester, USA; 3 Division of Hematology, Department of Medicine, Mayo Clinic, Rochester, MN USA; 4 Division of Hematology/Oncology, University of Arkansas for Medical Sciences, Little Rock, AR USA; 5 Division of Hematology, Department of Medicine, Mayo Clinic, Phoenix, AZ USA; 6 Division of Hematology & HSCT, Department of Oncology, King Abdulaziz Medical City, Riyadh, Saudi Arabia

**Keywords:** Myelodysplastic syndromes, Azacitidine, Decitabine, Network meta-analysis

## Abstract

**Background:**

Hypomethylating agents (HMA), azacitidine, and decitabine are frequently used in the management of myelodysplastic syndromes (MDS). However, there are no clinical trials that have directly compared these agents. We conducted a systematic review and indirectly compared the efficacy of azacitidine to decitabine in MDS.

**Methods:**

We conducted a comprehensive search of several databases (MEDLINE, EMBASE, Cochrane Central Register of Controlled Trials, and Scopus) through June 28, 2018, without language or time restrictions. Studies were screened by two independent reviewers, and differences were resolved by consensus. The fixed effect model and adjusted indirect comparison methods were used to pool relative risks (RR) of major outcomes of interest (mortality, response rate, quality of life, hematologic improvement, hospitalization, leukemia transformation, transfusion independence).

**Results:**

Only four trials met the eligibility criteria. Two trials compared azacitidine to the best supportive care (BSC) and included 549 patients, and the other two compared decitabine to BSC and included 403 patients. The risk of bias was unclear overall. Compared to BSC, azacitidine was significantly associated with lower mortality (RR = 0.83, 95% CI 0.74–0.94, *I*^2^ = 89%) whereas decitabine did not significantly reduce mortality (RR = 0.88, 95% CI 0.77–1.00, *I*^2^ = 53%). Both drugs were associated with higher partial and complete response compared to BSC. Indirect comparisons were not statistically significant for all the studied outcomes, except for complete response where azacitidine was less likely to induce complete response compared to decitabine (RR = 0.11, 95% CI = 0.01–0.86, very low-certainty evidence).

**Conclusions:**

Azacitidine and decitabine are both associated with improved outcomes compared to BSC. The available indirect evidence comparing the two agents warrants very low certainty and cannot reliably confirm the superiority of either agent. Head-to-head trials are needed. In the meantime, the choice of agent should be driven by patient preferences, adverse effects, drug availability, and cost.

**Electronic supplementary material:**

The online version of this article (10.1186/s13643-018-0805-7) contains supplementary material, which is available to authorized users.

## Background

Myelodysplastic syndromes (MDS) are a heterogeneous group of bone marrow disorders characterized by ineffective hematopoiesis of clonal stem cells and are distinguished by dysplasia in one or more hematopoietic cell lineages [1, 2]. Such disorders typically affect the elderly, with the majority of patients succumbing to their disease due to complications of bone marrow failure rather than transformation to acute leukemia [3]. The revised edition of the World Health Organization (WHO) classification of hematopoietic neoplasms included six general entities of MDS based upon a combination of clinical, morphologic, immunophenotypic, and genetic features [4].

Immediate treatment of MDS is indicated in patients with symptomatic cytopenias, which includes most patients with high- or very high-risk MDS. Panel of experts from the European LeukemiaNet and National Comprehensive Cancer Network (NCCN) have issued guidelines on optimal treatment strategies guided by risk stratification and patient’s performance status [5]. There are a number of prognostic risk scores that can be used to stratify the corresponding risk and accordingly assign appropriate therapy, among which is the International Prognostic Scoring System (IPSS) and its most recent revision, the revised IPSS (IPSS-R) which is widely used [6, 7]. In cases of high- and very high-risk status, intensive combination chemotherapy followed by allogeneic transplantation (HSCT) is recommended for patients with good performance status and available donors [8, 9]. Patients with high-risk disease but ineligible for intensive therapy can be offered hypomethylating agents (HMAs) among other options. The HMAs azacitidine and decitabine are pyrimidine nucleoside analogs of cytidine and are approved by the Food and Drug Administration (FDA) for the treatment of MDS.

Given that many MDS patients will be ineligible to receive intensive therapy or HSCT, the use of HMA remains a common strategy. Both azacitidine and decitabine have demonstrated superior response and longer time to leukemic transformation compared to supportive care alone, and either drug may be used in high-risk patients not fit for intensive therapy [10, 11]. However, these agents have not been compared directly in a randomized fashion, and the choice of therapy in this scenario depends largely on the experiences and preferences of the treating physician. In this study, our aim is to conduct a systematic review and network meta-analysis to compare the efficacy of azacitidine to decitabine in patients with MDS.

## Methods

The protocol of this study was developed a priori. We reported this systematic review according to the Preferred Reporting Items for Systematic Reviews and Meta-Analyses (PRISMA) statements [12]. The protocol of this systematic review has not been registered with PROSPERO.

### Eligibility criteria

We only included in this systematic review randomized controlled trials (RCTs) that investigated adults diagnosed with myelodysplastic syndromes and treated by one of the HMAs (azacitidine or decitabine) and compared them to placebo or standard supportive care, or compared the two drugs against each other. Trials had to report at least one of the following outcomes: mortality, response rate, quality of life, hematologic improvement, hospitalization, leukemia transformation, and transfusion independence. Death, complete and partial responses, and hematologic parameters were all defined according to the study protocols. We excluded non-randomized control trials focusing on comparing intervention regimens (weekly vs. monthly), and reviews.

### Data sources and search strategies

We performed a comprehensive electronic search of MEDLINE In-Process & Other Non-Indexed Citations, MEDLINE, EMBASE, Cochrane Central Register of Controlled Trials, Cochrane Database of Systematic Reviews, and Scopus through June 28, 2018, without language restrictions. The search strategy was designed and conducted by an experienced librarian with input from the study investigators. The detailed search strategy is available in the appendix (Additional file 1).

### Study selection

Two independent reviewers (JA, HA) screened all the titles and abstracts and assessed the eligibility of each article based on a priori chosen criteria. We retrieved the relevant references in full text and uploaded for full-text evaluation against eligibility criteria. Disagreements were resolved by consensus and with input from a third reviewer (AA).

### Data extraction

Two reviewers (JA, HA) independently extracted data using standardized, pilot-tested forms created in Microsoft Excel (2010). Disagreements were resolved by discussion between the two reviewers. We extracted the following variables from each study: study characteristics, participants’ description, intervention details, and outcomes of interest. Outcomes of interest were extracted as a number of patients with the outcome. We extracted data about the outcomes at the longest follow-up reported in the study.

### Methodologic quality and risk of bias

We used the Cochrane Collaboration’s tool for assessing the risk of bias in the trials [13], which included the following domains: random sequence generation, allocation concealment, blinding, incomplete outcome data, and selective outcome reporting. Using the Cochrane risk of bias assessment tool, the risk of bias could be assessed as low, unclear, or high. Two reviewers (JA, HA) independently assessed the risk of bias in each study. Any conflicts were resolved by consensus.

### Analysis

All analyses were based on the intention-to-treat (ITT) principle. We calculated relative risks (RRs) with associated 95% confidence intervals (95% CI) estimated using the binomial distribution. Continuity correction of 0.5 was used when the number of events was zero. Due to the heterogeneity of settings in which the trials were conducted, we used the method of Mantel and Haenszel fixed effects models to pool relative risks (RRs) because the number of studies is less than 3 [14]. We used *I*^2^ to evaluate the heterogeneity across studies by each outcome. *I*^2^ > 50% suggests substantial heterogeneity. Continuity correction of 0.5 was used when needed.

The two-step approach using the adjusted indirect comparisons was used to estimate RR for indirect comparisons between azacitidine and decitabine [15]. We also conducted random effects meta-regression using a frequentist consistency model as described by Ian White to generate ranking probabilities and the surface under the cumulative ranking curve (SUCRA) [16, 17]. All statistical analyses were conducted using STATA, version 15 (StataCorp LP, College Station, TX).

### Subgroup analysis

We planned to evaluate the outcomes separately in low-risk and high-risk patients (we defined high risk as bone marrow blast of 5% or above (refractory anemia with excess blasts (RAEB)-1 or RAEB2)).

### Sensitivity analysis

We planned to evaluate whether our conclusions would differ if a different analysis outcome measure was used (odds ratios vs. relative risks) and whether a different meta-analysis model was used.

### Certainty in the evidence

We evaluated the certainty in evidence (also called quality of evidence) using the GRADE (Grading of Recommendations, Assessment, Development and Evaluation) approach. Randomized trials warrant high certainty but can be downgraded for methodological limitations (risk of bias), imprecision, indirectness, inconsistency, and publication bias [18].

## Results

### Study selection

**Fig. 1:**
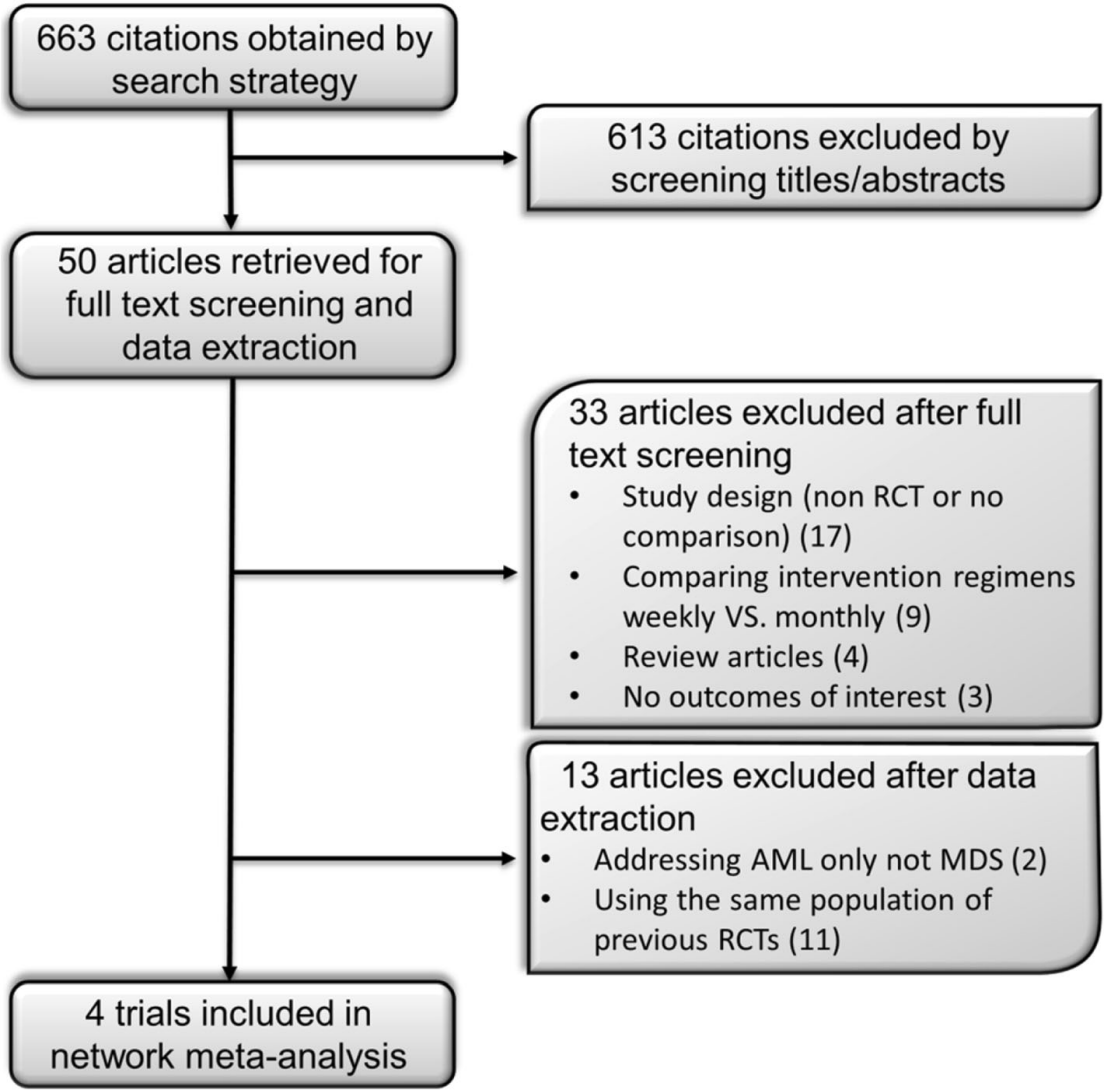
Flowchart of study selection process

**Fig. 2:**
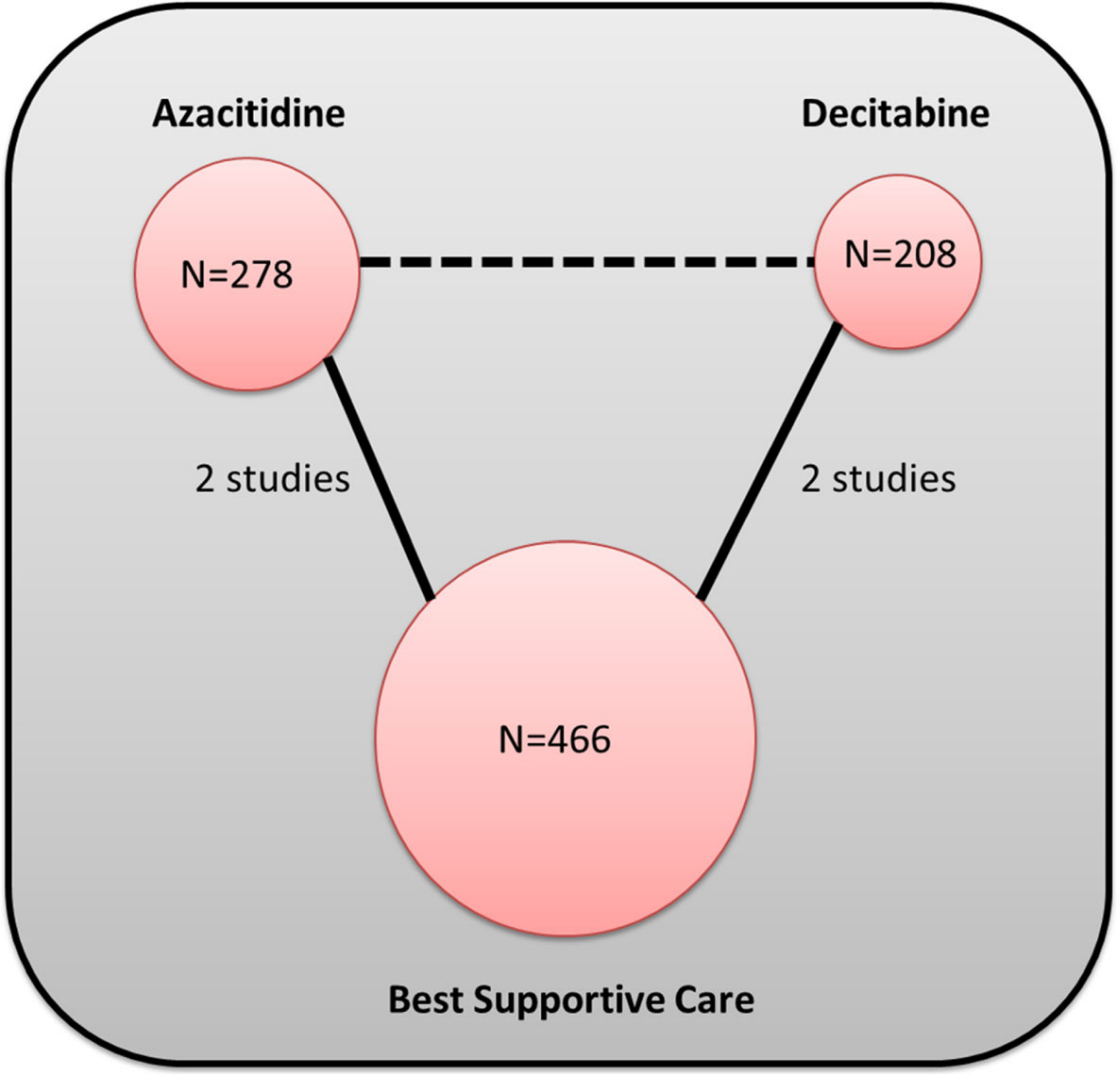
Network plot of the MDS network. Nodes are weighted according to the total number of patients in the included studies. Solid lines represent direct evidence. The dashed line represents indirect evidence

The electronic search strategy identified 663 citations before removal of duplicates. We excluded 613 articles during the abstract and full text screening process. Four trials met the selection criteria after data extraction and included in network meta-analysis. The PRISMA flow chart of the selection process is depicted in Fig. 1. The network plot of the MDS network is provided in Fig. 2.

### Study characteristics

The four RCTs are reported in 952 patients with age range 31–92 years old. The percentage of the patients with intermediate-2 and high-risk myelodysplastic syndrome (based on the International Prognostic Scoring System (IPSS)) was higher in decitabine studies (82.21% and 83.08% in decitabine and BSC, respectively) compared to azacitidine studies (62.59% and 61.26% in azacitidine and BSC, respectively). Two RCTs compared azacitidine (75 mg/m^2^/day SC × 7 days) to the best supportive care (BSC) with additional low-dose cytarabine and intensive chemotherapy in one of the trials [10] and included 549 patients (278 azacitidine and 271 BSC; age average 69; range 31–92 years old). The other two RCTs compared decitabine (15 mg/m^2^ IV q 8 h × 9) to the BSC and included 403 patients (208 decitabine and 195 BSC; age average 69–70; range 60–90 years old). The characteristics and citations of the included studies are summarized in Table 1.

**Table 1:** Trials characteristics

Author, year	Intervention (dose, schedule)	Comparison (description)	Patients (*N*)	Age (years)	Male (%)	IPSS classification intervention	IPSS classification comparison	Follow-up (months)
Azacitidine								
Fenaux, 2009 [10]	Azacitidine: subcutaneously 75 mg/m^2^/day for 7 days Q 28 days for at least six cycles	BSC, low-dose Ara-C 20 mg/m^2^, intensive chemotherapy, or mitoxantrone	Intervention: 179 Comparison: 179	Intervention: 69 (42–83) Comparison: 70 (38–88)	NR	Intermediate-1: 5 (3%) Intermediate-2: 76 (43%) High: 82 (46%)	Intermediate-1: 13 (7%) Intermediate-2: 70 (39%) High: 85 (48%)	42
Silverman, 2002 [19]	Azacitidine: subcutaneously 75 mg/m^2^/day for 7 days Q 28 days for at least six cycles	Supportive care with antibiotics and transfusions	Intervention: 99 Comparison: 92	Intervention: 69 (31–92) Comparison: 67 (35–88)	Intervention: 71.05 Comparison: 63.27	Low*: 2 (2%) Intermediate-1: 21 (26%) Intermediate-2: 9 (11%) High: 7 (9%)	Low*: 5 (6%) Intermediate-1: 16 (20%) Intermediate-2: 13 (16%) High: 8 (10%)	53
Decitabine								
Kantarjian, 2006 [21]	Decitabine: intravenously 15 mg/m^2^ Q 8 h for 3 days, every 6 weeks	BSC	Intervention: 89 Comparison: 81	Intervention: 70 (65–76) Comparison: 70 (62–74)	Intervention: 66.29 Comparison: 70.37	Intermediate-1: 28 (31%) Intermediate-2: 38 (43%) High: 23 (26%)	Intermediate-1: 24 (30%) Intermediate-2: 36 (44%) High: 21 (26%)	30
Lubbert, 2011 [11]	Decitabine: intravenously 15 mg/m^2^ Q 8 h for 3 days, every 6 weeks	BSC	Intervention: 119 Comparison: 114	Intervention: 69 (60–90) Comparison: 70 (60–86)	Intervention: 63.87 Comparison: 64.04	Intermediate-1: 8 (6.7%) Intermediate-2: 64 (53.8%) High: 46 (38.7%)	Intermediate-1: 8 (7%) Intermediate-2: 63 (55.3%) High: 42 (36.8%)	30

*BSC* best supportive care, *IPSS* International Prognostic Scoring System, *N* total number of patients

*Data reported on 81 patients

### Outcomes

Compared to BSC, azacitidine significantly reduced mortality (RR = 0.83, 95% CI 0.74–0.94, *p* = 0.002, *I*^2^ = 89.3%) (Additional file 1: Figure S1) whereas the effect of decitabine did not reach statistical significance (RR = 0.88, 95% CI 0.77–1.001, *p* = 0.053, *I*^2^ = 53.0%) (Additional file 1: Figure S2). Both drugs were superior to BSC in terms of partial and complete response. Head-to-head comparisons were not statistically significant for all the studied outcomes, except for the outcome of complete response where low-certainty evidence suggested that azacitidine-treated patients were less likely to have complete response compared to decitabine (RR = 0.11, 95% CI = 0.01, 0.86, *p* = 0.04) (Fig. 4).

Median time to acute leukemia transformation reported in two RCTs [10, 19] compared azacitidine to BSC and ranged from 17.8 to 21 vs. 11.5 to 12 months, respectively. In one RCT [10], 50 (45%) of 111patients who were treated with azacitidine became transfusion independent compared to 13 (11.4%) of 114 patients in the BSC group. SUCRA analysis slightly favored azacitidine over decitabine in terms of improving the overall survival (68.4% vs. 65.3%). Data and ranking figures are in the appendix.

The data on quality of life and hospitalization were not reported. Data were insufficient to conduct subgroup analyses based on bone marrow blast percentage. The conclusions did not differ when odds ratios were used instead of RR (appendix, Additional file 1). Random effects models using DerSimonian and Laird or Hartung-Knapp-Sidik-Jonkman estimators made the estimates imprecise due to the instability of between-study variance.

### Risk of bias

**Fig. 3:**
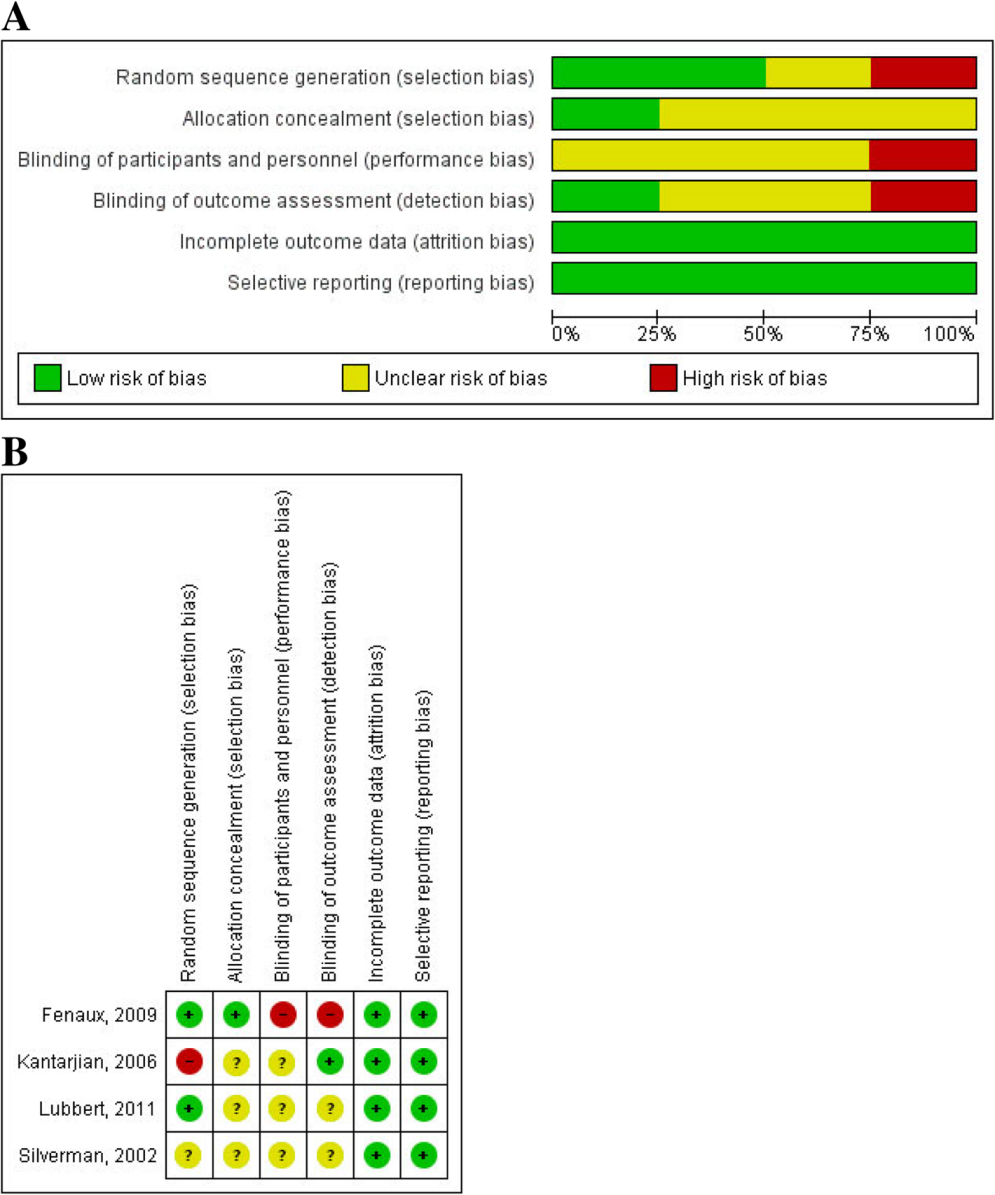
**a** Risk of bias graph. Review authors’ judgments about each risk of bias item presented as percentages across all included studies. **b** Risk of bias summary. Review authors’ judgments about each risk of bias item for each included study

Overall, the risk of bias within studies ranged from unclear to high. Random sequence generation was adequate in two trials, whereas allocation concealment was achieved only in one trial and blinding of outcome assessor in another one. However, all trials are low risk in regard to incomplete outcome data and selective reporting domains. The risk of bias graph and summary are provided in Fig. 3.

### Certainty in evidence

**Fig. 4:**
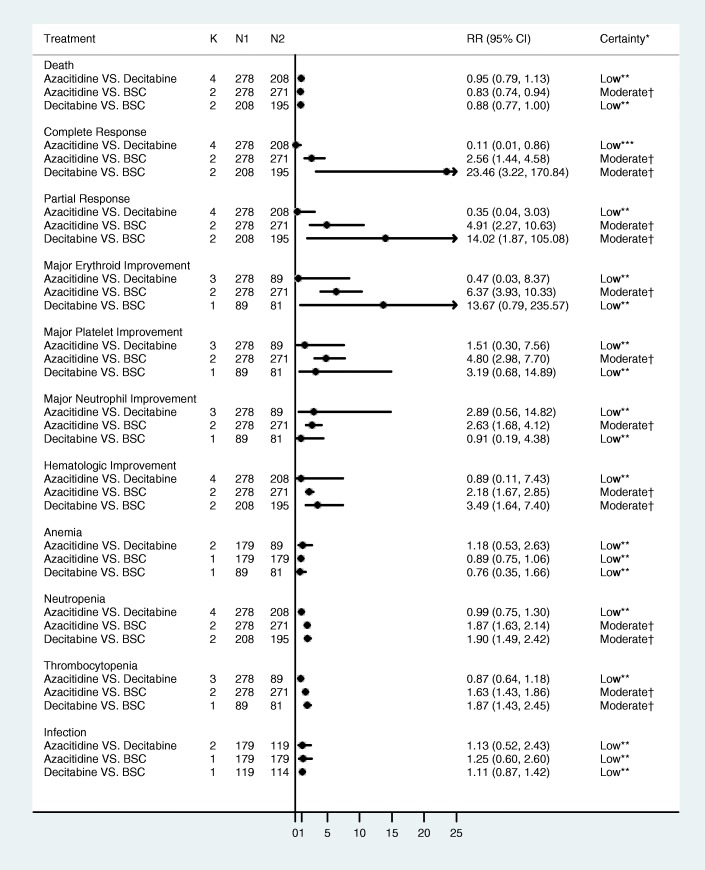
Forest plot represents the direct and indirect comparison relative risks and associated 95% confidence intervals (CI; horizontal lines) of death, complete and partial responses, and hematologic parameters for azacitidine group compared to the best supportive care group. Comparisons are direct except azacitidine vs decitabine. BSC, best supportive care; K, total number of RCTs; N, total number of patients. *Certainty: certainty in evidence (also called quality of evidence). **Rated down for imprecision (confidence intervals that include appreciable benefits and harms) and methodological limitation (unclear risk of bias). ***Rated down due to imprecision (small number of events, 14 patients achieving complete response in BSC arm in one trial) and rated down for methodological limitation (unclear risk of bias). ^†^Rated down due to methodological limitation (unclear risk of bias). It is also plausible to rate down for some imprecision due to the overall small sample size

For direct comparisons, we were unable to explore publication bias (two of the certainty domains). The risk of bias was unclear in all of the studied outcomes, which led to downgrading certainty. The certainty in evidence supporting the effect of azacitidine or decitabine compared to best medical care was low or moderate for the various outcomes (see Fig. 4 and Additional file 1 evidence profiles in the appendix). Indirect head-to-head comparison generated estimates that warranted low certainty due to imprecision and risk of bias. Network consistency could not be evaluated based on network geometry (no closed loops).

## Discussion

Only three disease-modifying agents have been approved for the treatment of MDS, namely, lenalidomide (in 5q deletion MDS patients) and the HMAs, azacitidine and decitabine [10, 19–21]. In addition, immune-suppressive therapy and erythropoietin-stimulating agents can be used in lower-risk MDS cases [22–24]. Autologous stem cell transplantation (ASCT) remains the only potentially curative method as none of the above agents can achieve a cure [25]. Clinical trials are needed, and enrollment is recommended for most MDS patients.

In this systematic review and network meta-analysis, we attempted to evaluate the comparative-effectiveness of azacitidine and decitabine in patients with MDS. We have demonstrated that both azacitidine and decitabine are likely to have better outcomes compared to the best supportive care in terms of all-cause mortality, overall response, and hematologic improvements. Based on the available evidence, the indirect comparisons between azacitidine and decitabine show no superiority of one agent over the other.

These findings should be interpreted in the context of the presentation of each patient and their prognostic risk classification. HMAs remain the preferred treatment in those with intermediate-2/high IPSS risk MDS patients to be followed by ASCT if the patient qualifies and a donor is found, although, in some instances, direct ASCT or prior intensive chemotherapy is considered depending on the karyotype and blast percentage. It could be argued based on our findings; if debulking prior to ASCT is needed, then treatment with decitabine is favored as it has a higher chance to achieve remission. Azacitidine rendered a 7% complete remission rate and 16% partial remission rate, with an overall remission rate of 23% in the CALGB 9221 [19]. Decitabine had a 13% complete remission rate and 6% partial rate, with an overall remission rate of 19% as reported by the European Organisation for Research and Treatment of Cancer Leukemia Group and the German MDS Study Group [11]. Additionally, both studies quoted used the every 8-h schedule of decitabine and not the daily schedule for 5 days based on more recent data [26]. On the other hand, most studies in MDS did not show any correlation between response and survival; hence, such a decision of which HMA to use should not rely only on complete remission rate [27].

A previous systematic review and meta-analysis published in 2010 [28] compared both HMAs agents (azacitidine and decitabine) to conventional care in patients with MDS and has identified four trials (two per each agent). Meta-analysis of all four trials showed that HMAs overall improved survival and time to transformation or death. These results are consistent with our findings although we have addressed additional outcomes. Furthermore, our aim was to compare the two agents, hoping to generate inferences for patients and clinicians facing the choice of using one of the two agents. This dilemma is common in oncology practice given that many MDS patients will be ineligible to receive intensive therapy or HSCT and require one of these two HMAs.

### Limitations

Given the limited number of trials investigating each agent, heterogeneity, network consistency, and publication bias could not be adequately assessed. The size of the body of evidence remains small. Optimally, a risk stratification model could be developed to capture the effects of HMAs in the different risk groups. This could not be obtained by performing subgroup analysis due to the paucity of data. The analysis was not robust to sensitivity analyses based on meta-analysis model choice.

## Conclusions

Azacitidine and decitabine are both likely to be superior to BSC. The available indirect evidence comparing the two agents warrants very low certainty and cannot reliably confirm the superiority of either agent. Head-to-head trials are needed to provide a better understanding of the relative effectiveness of azacitidine and decitabine. In the meantime, the choice of agent should be driven by patients’ preferences, drug availability and adverse effects, and cost.

## Additional file

Additional file 1: Appendix. (DOCX 1773 kb)

## Abbreviations

ASCT: Autologous stem cell transplantation
BSC: Best supportive care
HMA: Hypomethylating agent
MDS: Myelodysplastic syndrome
RCT: Randomized controlled trial
