# Supplementary material for: A systematic review and network meta-analysis comparing azacitidine and decitabine for the treatment of myelodysplastic syndrome
Source: Syst Rev. 2018 Sep 19;7:144. doi: 10.1186/s13643-018-0805-7 (PMC6145118; doi:10.1186/s13643-018-0805-7)
Supplement: Supplementary file 1 — Appendix. (DOCX 1773 kb) [file 13643_2018_805_MOESM1_ESM.docx]

| **A Systematic Review and Network Meta-analysis Comparing Azacitidine and Decitabine for the Treatment of Myelodysplastic Syndrome (Appendix)** |
| --- |
|  |
| Jehad Almasri, M.D. Hassan B. Alkhateeb, M.D. Belal Firwana, M.D. Mohamad Bassam Sonbol, M.D. Moussab Damlaj, M.D. Zhen Wang,Ph.D. M. Hassan Murad, M.D. M.P.H. Aref Al-Kali, M.D. |

Table of Contents

[*Supplemental Figures* 3](#_Toc516249148)

[*Figure S1: Azacitidine vs. Best Supportive Care (Direct Evidence-RR)* 3](#_Toc516249149)

[*Figure S2: Decitabine vs. Best Supportive Care (Direct Evidence-RR)* 4](#_Toc516249150)

[*Figure S3: Azacitidine vs. Best Supportive Care (Direct Evidence-OR)* 5](#_Toc516249151)

[*Figure S4: Decitabine vs. Best Supportive Care (Direct Evidence-OR)* 6](#_Toc516249152)

[*Figure S5: The surface under the cumulative ranking curves for survival outcome* 7](#_Toc516249153)

[*The surface under the cumulative ranking curve values for survival outcome* 8](#_Toc516249154)

[*Certainty in the evidence tables (Evidence profiles)* 8](#_Toc516249155)

[*Azacitidine compared to BSC for adults diagnosed with MDS* 8](#_Toc516249156)

[*Decitabine compared to BSC for adults diagnosed with MDS* 14](#_Toc516249157)

[*Azacitidine compared to decitabine for adults diagnosed with MDS* 19](#_Toc516249158)

[*Actual Search Strategies:* 24](#_Toc516249159)

# *Supplemental Figures*

## *Figure S1: Azacitidine vs. Best Supportive Care (Direct Evidence-RR)*

Forest plot represents the direct comparison relative risks and associated 95% confidence intervals (CI; horizontal lines) of death, complete and partial responses, and hematologic parameters for azacitidine group compared to best supportive care group. N: Total number of patients; n: Total number of events.

## *Figure S2: Decitabine vs. Best Supportive Care (Direct Evidence-RR)*

Forest plot represents the direct comparison relative risks and associated 95% confidence intervals (CI; horizontal lines) of death, complete and partial responses, and hematologic parameters for decitabine group compared to best supportive care group. N: Total number of patients; n: Total number of events.

## *Figure S3: Azacitidine vs. Best Supportive Care (Direct Evidence-OR)*

Forest plot represents the direct comparison odds ratios and associated 95% confidence intervals (CI; horizontal lines) of death, complete and partial responses, and hematologic parameters for azacitidine group compared to best supportive care group. N: Total number of patients; n: Total number of events.

## *Figure S4: Decitabine vs. Best Supportive Care (Direct Evidence-OR)*

Forest plot represents the direct comparison odds ratios and associated 95% confidence intervals (CI; horizontal lines) of death, complete and partial responses, and hematologic parameters for decitabine group compared to best supportive care group. N: Total number of patients; n: Total number of events.

## *Figure S5: The surface under the cumulative ranking curves for survival outcome*

**
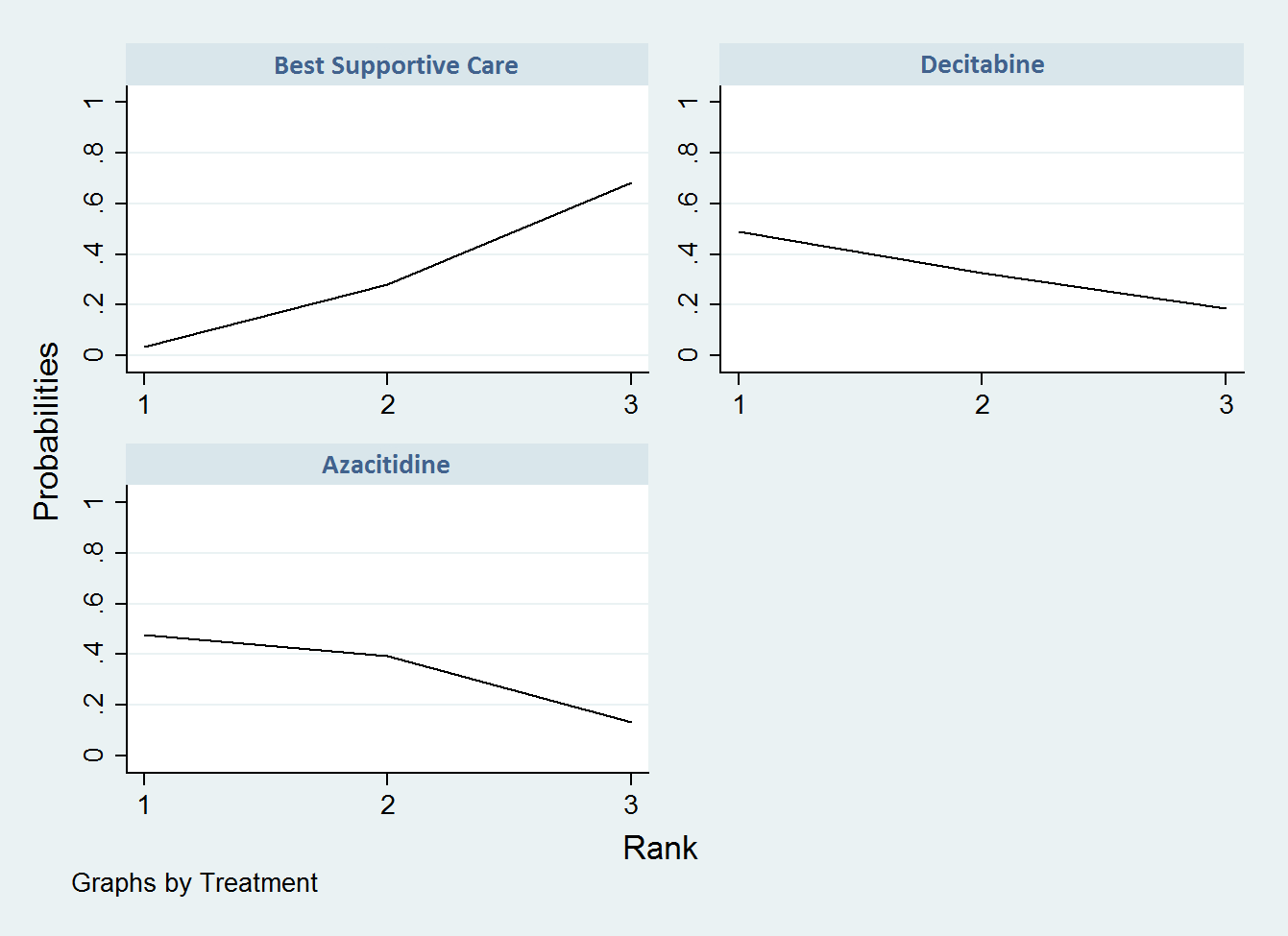
**

# *The surface under the cumulative ranking curve values for survival outcome*

| Treatment | SUCRA* | Probability as Best | Mean Rank |
| --- | --- | --- | --- |
| Best Supportive Care | 16.3 | 2.6 | 2.7 |
| Decitabine | 65.3 | 49.5 | 1.7 |
| Azacitidine | 68.4 | 47.9 | 1.6 |

***** Surface under the cumulative ranking curve

# *Certainty in the evidence tables (Evidence profiles)*

## *Azacitidine compared to BSC for adults diagnosed with MDS*

| **Certainty assessment** | | | | | | | **№ of patients** | | **Effect** | | **Certainty** | **Importance** |
| --- | --- | --- | --- | --- | --- | --- | --- | --- | --- | --- | --- | --- |
| **№ of studies** | **Study design** | **Risk of bias** | **Inconsistency** | **Indirectness** | **Imprecision** | **Other considerations** | **azacitidine** | **BSC** | **Relative (95% CI)** | **Absolute (95% CI)** |
| Death (follow up: median 48 months) | | | | | | | | | | | | |
| 2 | randomized trials | serious* | not serious | not serious | not serious | none | 166/278 (59.7%) | 193/271 (71.2%) | **RR 0.83** (0.74 to 0.94) | **121 fewer per 1,000** (from 43 fewer to 185 fewer) | ⨁⨁⨁◯ MODERATE | CRITICAL |
| Complete response (follow up: median 48 months) | | | | | | | | | | | | |
| 2 | randomized trials | serious* | not serious | not serious | not serious | none | 37/278 (13.3%) | 14/271 (5.2%) | **RR 2.56** (1.44 to 4.58) | **81 more per 1,000** (from 23 more to 185 more) | ⨁⨁⨁◯ MODERATE | CRITICAL |
| Partial response (follow up: median 48 months) | | | | | | | | | | | | |
| 2 | randomized trials | serious* | not serious | not serious | not serious | none | 37/278 (13.3%) | 7/271 (2.6%) | **RR 4.91** (2.27 to 10.63) | **101 more per 1,000** (from 33 more to 249 more) | ⨁⨁⨁◯ MODERATE | CRITICAL |
| Major erythroid improvement (follow up: median 48 months) | | | | | | | | | | | | |
| 2 | randomized trials | serious* | not serious | not serious | not serious | none | 113/278 (40.6%) | 17/271 (6.3%) | **RR 6.37** (3.93 to 10.33) | **337 more per 1,000** (from 184 more to 585 more) | ⨁⨁⨁◯ MODERATE | CRITICAL |
| Major platelet improvement (follow up: median 48 months) | | | | | | | | | | | | |
| 2 | randomized trials | serious* | not serious | not serious | not serious | none | 93/278 (33.5%) | 19/271 (7.0%) | **RR 4.79** (2.98 to 7.70) | **266 more per 1,000** (from 139 more to 470 more) | ⨁⨁⨁◯ MODERATE | CRITICAL |
| Major neutrophil improvement (follow up: median 48 months) | | | | | | | | | | | | |
| 2 | randomized trials | serious* | not serious | not serious | not serious | none | 65/278 (23.4%) | 24/271 (8.9%) | **RR 2.63** (1.68 to 4.12) | **144 more per 1,000** (from 60 more to 276 more) | ⨁⨁⨁◯ MODERATE | CRITICAL |
| Hematologic improvement (follow up: median 48 months) | | | | | | | | | | | | |
| 2 | randomized trials | serious* | not serious | not serious | not serious | none | 124/278 (44.6%) | 56/271 (20.7%) | **RR 2.18** (1.67 to 2.85) | **244 more per 1,000** (from 138 more to 382 more) | ⨁⨁⨁◯ MODERATE | CRITICAL |
| Anemia (follow up: median 42 months) | | | | | | | | | | | | |
| 1 | randomized trials | serious* | not serious | not serious | serious** | none | 100/179 (55.9%) | 112/179 (62.6%) | **RR 0.89** (0.75 to 1.06) | **69 fewer per 1,000** (from 156 fewer to 38 more) | ⨁⨁◯◯ LOW | CRITICAL |
| Neutropenia (follow up: median 48 months) | | | | | | | | | | | | |
| 2 | randomized trials | serious* | not serious | not serious | not serious | none | 239/278 (86.0%) | 126/271 (46.5%) | **RR 1.87** (1.63 to 2.14) | **405 more per 1,000** (from 293 more to 530 more) | ⨁⨁⨁◯ MODERATE | CRITICAL |
| Thrombocytopenia (follow up: median 48 months) | | | | | | | | | | | | |
| 2 | randomized trials | serious* | not serious | not serious | not serious | none | 218/278 (78.4%) | 132/271 (48.7%) | **RR 1.63** (1.43 to 1.86) | **307 more per 1,000** (from 209 more to 419 more) | ⨁⨁⨁◯ MODERATE | CRITICAL |
| Infection (follow up: median 42 months) | | | | | | | | | | | | |
| 1 | randomized trials | serious* | not serious | not serious | serious** | none | 15/179 (8.4%) | 12/179 (6.7%) | **RR 1.25** (0.60 to 2.60) | **17 more per 1,000** (from 27 fewer to 107 more) | ⨁⨁◯◯ LOW | CRITICAL |

CI: Confidence interval; RR: Risk ratio

*Inadequate allocation concealment and blinding in some trials

** Confidence intervals that includes appreciable benefits and harms

## *Decitabine compared to BSC for adults diagnosed with MDS*

| **Certainty assessment** | | | | | | | **№ of patients** | | **Effect** | | **Certainty** | **Importance** |
| --- | --- | --- | --- | --- | --- | --- | --- | --- | --- | --- | --- | --- |
| **№ of studies** | **Study design** | **Risk of bias** | **Inconsistency** | **Indirectness** | **Imprecision** | **Other considerations** | **decitabine** | **BSC** | **Relative (95% CI)** | **Absolute (95% CI)** |
| Death (follow up: median 30 months) | | | | | | | | | | | | |
| 2 | randomized trials | serious* | not serious | not serious | serious** | none | 108/208 (51.9%) | 117/195 (60.0%) | **RR 0.880** (0.770 to 1.001) | **72 fewer per 1,000** (from 1 more to 138 fewer) | ⨁⨁◯◯ LOW | CRITICAL |
| Complete response (follow up: median 30 months) | | | | | | | | | | | | |
| 2 | randomized trials | serious* | not serious | not serious | not serious | none | 24/208 (11.5%) | 0/195 (0.0%) | **RR 23.46** (3.22 to 170.84) | **0 fewer per 1,000** (from 0 fewer to 0 fewer) | ⨁⨁⨁◯ MODERATE | CRITICAL |
| Partial response (follow up: median 30 months) | | | | | | | | | | | | |
| 2 | randomized trials | serious* | not serious | not serious | not serious | none | 14/208 (6.7%) | 0/195 (0.0%) | **RR 14.02** (1.87 to 105.08) | **0 fewer per 1,000** (from 0 fewer to 0 fewer) | ⨁⨁⨁◯ MODERATE | CRITICAL |
| Major erythroid improvement (follow up: median 30 months) | | | | | | | | | | | | |
| 1 | randomized trials | serious* | not serious | not serious | serious** | none | 7/89 (7.9%) | 0/81 (0.0%) | **RR 13.67** (0.79 to 235.57) | **0 fewer per 1,000** (from 0 fewer to 0 fewer) | ⨁⨁◯◯ LOW | CRITICAL |
| Major platelet improvement (follow up: median 30 months) | | | | | | | | | | | | |
| 1 | randomized trials | serious* | not serious | not serious | serious** | none | 7/89 (7.9%) | 2/81 (2.5%) | **RR 3.19** (0.68 to 14.89) | **54 more per 1,000** (from 8 fewer to 343 more) | ⨁⨁◯◯ LOW | CRITICAL |
| Major neutrophil improvement (follow up: median 30 months) | | | | | | | | | | | | |
| 1 | randomized trials | serious* | not serious | not serious | serious** | none | 3/89 (3.4%) | 3/81 (3.7%) | **RR 0.91** (0.19 to 4.38) | **3 fewer per 1,000** (from 30 fewer to 125 more) | ⨁⨁◯◯ LOW | CRITICAL |
| Hematologic improvement (follow up: median 30 months) | | | | | | | | | | | | |
| 2 | randomized trials | serious* | not serious | not serious | not serious | none | 30/208 (14.4%) | 8/195 (4.1%) | **RR 3.49** (1.64 to 7.40) | **102 more per 1,000** (from 26 more to 263 more) | ⨁⨁⨁◯ MODERATE | CRITICAL |
| Anemia (follow up: median 30 months) | | | | | | | | | | | | |
| 1 | randomized trials | serious* | not serious | not serious | serious** | none | 10/89 (11.2%) | 12/81 (14.8%) | **RR 0.76** (0.35 to 1.66) | **36 fewer per 1,000** (from 96 fewer to 98 more) | ⨁⨁◯◯ LOW | CRITICAL |
| Neutropenia (follow up: median 30 months) | | | | | | | | | | | | |
| 2 | randomized trials | serious* | not serious | not serious | not serious | none | 101/208 (48.6%) | 49/195 (25.1%) | **RR 1.90** (1.49 to 2.42) | **226 more per 1,000** (from 123 more to 357 more) | ⨁⨁⨁◯ MODERATE | CRITICAL |
| Thrombocytopenia (follow up: median 30 months) | | | | | | | | | | | | |
| 1 | randomized trials | serious* | not serious | not serious | not serious | none | 72/89 (80.9%) | 35/81 (43.2%) | **RR 1.87** (1.43 to 2.45) | **376 more per 1,000** (from 186 more to 627 more) | ⨁⨁⨁◯ MODERATE | CRITICAL |
| Infection (follow up: median 30 months) | | | | | | | | | | | | |
| 1 | randomized trials | serious* | not serious | not serious | serious** | none | 66/119 (55.5%) | 57/114 (50.0%) | **RR 1.11** (0.87 to 1.42) | **55 more per 1,000** (from 65 fewer to 210 more) | ⨁⨁◯◯ LOW | CRITICAL |

CI: Confidence interval; RR: Risk ratio

*Inadequate allocation concealment and blinding in some trials

** Confidence intervals that includes appreciable benefits and harms

## *Azacitidine compared to decitabine for adults diagnosed with MDS*

| **Certainty assessment** | | | | | | | **№ of patients** | | **Effect** | | **Certainty** | **Importance** |
| --- | --- | --- | --- | --- | --- | --- | --- | --- | --- | --- | --- | --- |
| **№ of studies** | **Study design** | **Risk of bias** | **Inconsistency** | **Indirectness** | **Imprecision** | **Other considerations** | **azacitidine** | **decitabine** | **Relative (95% CI)** | **Absolute (95% CI)** |
| Death | | | | | | | | | | | | |
| 4 | randomized trials | serious* | not serious | not serious | serious** | none | 166/278 (59.7%) | 108/208 (51.9%) | **RR 0.95** (0.79 to 1.13) | **26 fewer per 1,000** (from 67 more to 109 fewer) | ⨁⨁◯◯ LOW | CRITICAL |
| Complete Response | | | | | | | | | | | | |
| 4 | randomized trials | serious* | not serious | not serious | serious† | none | 37/278 (13.3%) | 24/208 (11.5%) | **RR 0.11** (0.01 to 0.86) | **103 fewer per 1,000** (from 16 fewer to 114 fewer) | ⨁⨁◯◯ LOW | CRITICAL |
| Partial Response | | | | | | | | | | | | |
| 4 | randomized trials | serious* | not serious | not serious | serious** | none | 37/278 (13.3%) | 14/208 (6.7%) | **RR 0.35** (0.04 to 3.03) | **44 fewer per 1,000** (from 65 fewer to 137 more) | ⨁⨁◯◯ LOW | CRITICAL |
| Major Erythroid Improvement | | | | | | | | | | | | |
| 3 | randomized trials | serious* | not serious | not serious | serious** | none | 113/278 (40.6%) | 7/89 (7.9%) | **RR 0.47** (0.03 to 8.37) | **42 fewer per 1,000** (from 76 fewer to 580 more) | ⨁⨁◯◯ LOW | CRITICAL |
| Major Platelet Improvement | | | | | | | | | | | | |
| 3 | randomized trials | serious* | not serious | not serious | serious** | none | 93/278 (33.5%) | 7/89 (7.9%) | **RR 3.19** (0.68 to 14.89) | **172 more per 1,000** (from 25 fewer to 1,000 more) | ⨁⨁◯◯ LOW | CRITICAL |
| Major Neutrophil Improvement | | | | | | | | | | | | |
| 3 | randomized trials | serious* | not serious | not serious | serious** | none | 65/278 (23.4%) | 3/89 (3.4%) | **RR 2.89** (0.56 to 14.82) | **64 more per 1,000** (from 15 fewer to 466 more) | ⨁⨁◯◯ LOW | CRITICAL |
| Hematologic Improvement | | | | | | | | | | | | |
| 4 | randomized trials | serious* | not serious | not serious | serious** | none | 124/278 (44.6%) | 30/208 (14.4%) | **RR 0.89** (0.11 to 7.43) | **16 fewer per 1,000** (from 128 fewer to 927 more) | ⨁⨁◯◯ LOW | CRITICAL |
| Anemia | | | | | | | | | | | | |
| 2 | randomized trials | serious* | not serious | not serious | serious** | none | 100/179 (55.9%) | 10/89 (11.2%) | **RR 1.18** (0.53 to 2.63) | **20 more per 1,000** (from 53 fewer to 183 more) | ⨁⨁◯◯ LOW | CRITICAL |
| Neutropenia | | | | | | | | | | | | |
| 4 | randomized trials | serious* | not serious | not serious | serious** | none | 239/278 (86.0%) | 101/208 (48.6%) | **RR 0.99** (0.75 to 1.30) | **5 fewer per 1,000** (from 121 fewer to 146 more) | ⨁⨁◯◯ LOW | CRITICAL |
| Thrompocytopenia | | | | | | | | | | | | |
| 3 | randomized trials | serious* | not serious | not serious | serious** | none | 218/278 (78.4%) | 72/89 (80.9%) | **RR 0.87** (0.64 to 1.18) | **105 fewer per 1,000** (from 146 more to 291 fewer) | ⨁⨁◯◯ LOW | CRITICAL |
| Infection | | | | | | | | | | | | |
| 2 | randomized trials | serious* | not serious | not serious | serious** | none | 15/179 (8.4%) | 66/119 (55.5%) | **RR 1.13** (0.52 to 2.43) | **72 more per 1,000** (from 266 fewer to 793 more) | ⨁⨁◯◯ LOW | CRITICAL |

CI: Confidence interval; RR: Risk ratio

*Inadequate allocation concealment and blinding in some trials

** Confidence intervals that includes appreciable benefits and harms

† Imprecision due to small number of events and CI boundary that is close to the null

# *Actual Search Strategies:*

Ovid

Database(s): Embase 1988 to 2017 Week 07, EBM Reviews - Cochrane Central Register of Controlled Trials January 2017, Ovid MEDLINE(R) Epub Ahead of Print, In-Process & Other Non-Indexed Citations, Ovid MEDLINE(R) Daily and Ovid MEDLINE(R) 1946 to Present
Search Strategy:

| **#** | **Searches** | **Results** |
| --- | --- | --- |
| 1 | exp Myelodysplastic Syndromes/ | 54432 |
| 2 | ("5q-syndrome*" or aCML or "atypical chronic myelogenous leukaemia*" or "atypical chronic myelogenous leukemia*" or "atypical chronic myeloid leukaemia*" or "atypical chronic myeloid leukemia*" or "bone marrow dysplasia*" or "chronic myelomonocytic leukaemia*" or "chronic myelomonocytic leukemia*" or CMML or CMMoL or "cold paroxysmal hemoglobinuria" or "dysmyelopoietic syndrome*" or "hematopoetic myelodysplasia**" or JMML or "juvenile myelomonocytic leukaemia*" or "juvenile myelomonocytic leukemia*" or "marchiafava micheli syndrome*" or myelodysplasia* or "myelodysplastic myeloproliferative disease" or "myelodysplastic myeloproliferative disorder" or "myelodysplastic syndrome*" or "nocturnal paroxysmal hemoglobinuria" or "oligoblastic leukaemia*" or "oligoblastic leukemia*" or "paroxysmal hemoglobinuria" or "paroxysmal nocturnal hemoglobinuria" or RAEB or "RAEB 1" or "RAEB 2" or "RAEB I" or "RAEB II" or RAEBt or "RAEB-t" or RCMD or "RCMD-RS" or "refractory anaemia*" or "refractory anemia*" or "refractory cytopaenia with multilineage dysplasia*" or "refractory cytopaenia with multilineage dysplasia* and ringed sideroblast*" or "refractory cytopenia with multilineage dysplasia*" or "refractory cytopenia with multilineage dysplasia* and ringed sideroblast*" or "sideroblastic anaemia*" or "sideroblastic anemia*").mp. | 71353 |
| 3 | 1 or 2 | 73514 |
| 4 | exp azacitidine/ | 15641 |
| 5 | exp 5 aza 2' deoxycytidine/ | 1834 |
| 6 | ((hypomethylating adj (agent* or drug* or medication*)) or "2 deoxy 5 azacytidine" or "5 aza 2 deoxycytidine" or "5 aza 2 desoxycytidine" or "5 azadeoxycytidine" or "5 azadesoxycytidine" or azacitidin or azacitidine or azacyd or azacytidin or azacytidine or dacogen or decitabine or ladakamycin or mylosar or "nsc 127716" or nsc102816 or "nsc-102816" or nsc127716 or vidaza).mp. | 27658 |
| 7 | 4 or 5 or 6 | 27658 |
| 8 | 3 and 7 | 6480 |
| 9 | exp Randomized Controlled Trial/ | 911996 |
| 10 | exp triple blind procedure/ | 204 |
| 11 | exp Double-Blind Method/ | 393652 |
| 12 | exp Single-Blind Method/ | 68979 |
| 13 | exp latin square design/ | 570 |
| 14 | exp Placebos/ | 344610 |
| 15 | exp Placebo Effect/ | 10229 |
| 16 | ((randomized adj3 study) or (randomized adj3 trial) or (randomised adj3 study) or (randomised adj3 trial) or "pragmatic clinical trial" or (doubl* adj blind*) or (doubl* adj mask*) or (singl* adj blind*) or (singl* adj mask*) or (tripl* adj blind*) or (tripl* adj mask*) or (trebl* adj blind*) or (trebl* adj mask*) or "latin square" or placebo* or nocebo* or random*).mp,pt. | 3385094 |
| 17 | or/9-16 | 3385094 |
| 18 | 8 and 17 | 839 |
| 19 | from 8 keep 3138-4154 | 1017 |
| 20 | limit 19 to (randomized controlled trial or pragmatic clinical trial) [Limit not valid in Embase,CCTR; records were retained] | 20 |
| 21 | 18 or 20 | 839 |
| 22 | limit 21 to (book or book series or editorial or erratum or letter or note or addresses or autobiography or bibliography or biography or comment or dictionary or directory or interactive tutorial or interview or lectures or legal cases or legislation or news or newspaper article or overall or patient education handout or periodical index or portraits or published erratum or video-audio media or webcasts) [Limit not valid in Embase,CCTR,Ovid MEDLINE(R),Ovid MEDLINE(R) Daily Update,Ovid MEDLINE(R) In-Process,Ovid MEDLINE(R) Publisher; records were retained] | 44 |
| 23 | 21 not 22 | 795 |
| 24 | limit 23 to ("all adult (19 plus years)" or "young adult (19 to 24 years)" or "adult (19 to 44 years)" or "young adult and adult (19-24 and 19-44)" or "middle age (45 to 64 years)" or "middle aged (45 plus years)" or "all aged (65 and over)" or "aged (80 and over)") [Limit not valid in Embase,CCTR; records were retained] | 728 |
| 25 | limit 24 to (adult <18 to 64 years> or aged <65+ years>) [Limit not valid in CCTR,Ovid MEDLINE(R),Ovid MEDLINE(R) Daily Update,Ovid MEDLINE(R) In-Process,Ovid MEDLINE(R) Publisher; records were retained] | 330 |
| 26 | limit 23 to ("all infant (birth to 23 months)" or "all child (0 to 18 years)" or "newborn infant (birth to 1 month)" or "infant (1 to 23 months)" or "preschool child (2 to 5 years)" or "child (6 to 12 years)" or "adolescent (13 to 18 years)") [Limit not valid in Embase,CCTR; records were retained] | 668 |
| 27 | limit 26 to (embryo or infant or child or preschool child <1 to 6 years> or school child <7 to 12 years> or adolescent <13 to 17 years>) [Limit not valid in CCTR,Ovid MEDLINE(R),Ovid MEDLINE(R) Daily Update,Ovid MEDLINE(R) In-Process,Ovid MEDLINE(R) Publisher; records were retained] | 116 |
| 28 | 27 not 25 | 5 |
| 29 | 23 not 28 | 790 |
| 30 | remove duplicates from 29 | 583 |

Ovid

1. TITLE-ABS-KEY("5q-syndrome*" OR aCML OR "atypical chronic myelogenous leukaemia*" OR "atypical chronic myelogenous leukemia*" OR "atypical chronic myeloid leukaemia*" OR "atypical chronic myeloid leukemia*" OR "bone marrow dysplasia*" OR "chronic myelomonocytic leukaemia*" OR "chronic myelomonocytic leukemia*" OR CMML OR CMMoL OR "cold paroxysmal hemoglobinuria" OR "dysmyelopoietic syndrome*" OR "hematopoetic myelodysplasia**" OR JMML OR "juvenile myelomonocytic leukaemia*" OR "juvenile myelomonocytic leukemia*" OR "marchiafava micheli syndrome*" OR myelodysplasia* OR "myelodysplastic myeloproliferative disease" OR "myelodysplastic myeloproliferative disorder" OR "myelodysplastic syndrome*" OR "nocturnal paroxysmal hemoglobinuria" OR "oligoblastic leukaemia*" OR "oligoblastic leukemia*" OR "paroxysmal hemoglobinuria" OR "paroxysmal nocturnal hemoglobinuria" OR RAEB OR "RAEB 1" OR "RAEB 2" OR "RAEB I" OR "RAEB II" OR RAEBt OR "RAEB-t" OR RCMD OR "RCMD-RS" OR "refractory anaemia*" OR "refractory anemia*" OR "refractory cytopaenia with multilineage dysplasia*" OR "refractory cytopaenia with multilineage dysplasia* and ringed sideroblast*" OR "refractory cytopenia with multilineage dysplasia*" OR "refractory cytopenia with multilineage dysplasia* and ringed sideroblast*" OR "sideroblastic anaemia*" OR "sideroblastic anemia*")
2. TITLE-ABS-KEY((hypomethylating W/1 (agent* or drug* or medication*)) OR "2 deoxy 5 azacytidine" OR "5 aza 2 deoxycytidine" OR "5 aza 2 desoxycytidine" OR "5 azadeoxycytidine" OR "5 azadesoxycytidine" OR azacitidin OR azacitidine OR azacyd OR azacytidin OR azacytidine OR dacogen OR decitabine OR ladakamycin OR mylosar OR "nsc 127716" OR nsc102816 OR "nsc-102816" OR nsc127716 OR vidaza)
3. TITLE-ABS-KEY((randomized W/3 study) OR (randomized W/3 trial) OR (randomised W/3 study) OR (randomised W/3 trial) OR "pragmatic clinical trial" OR (doubl* W/1 blind*) OR (doubl* W/1 mask*) OR (singl* W/1 blind*) OR (singl* W/1 mask*) OR (tripl* W/1 blind*) OR (tripl* W/1 mask*) OR (trebl* W/1 blind*) OR (trebl* W/1 mask*) OR "latin square" OR placebo* OR nocebo* OR random*)
4. 1 and 2 and 3
5. TITLE(newborn* or neonat* or infant* or child* or adolescent* or paediatric* or pediatric*)
6. TITLE-ABS-KEY(adult or adults or "middle age" or "middle aged" or elderly or geriatric*)
7. 5 and not 6
8. 4 and not 7
9. DOCTYPE(le) OR DOCTYPE(ed) OR DOCTYPE(bk) OR DOCTYPE(er) OR DOCTYPE(no) OR DOCTYPE(sh)
10. 8 and not 9
11. PMID(0*) OR PMID(1*) OR PMID(2*) OR PMID(3*) OR PMID(4*) OR PMID(5*) OR PMID(6*) OR PMID(7*) OR PMID(8*) OR PMID(9*)
12. 10 and not 11
